# Scopolamine augmentation of a newly initiated escitalopram treatment for major depressive disorder: study protocol for a randomized controlled trial

**DOI:** 10.1186/s13063-018-3132-3

**Published:** 2019-01-09

**Authors:** Jingjing Zhou, Weiwei Wang, Jian Yang, Xuequan Zhu, Lei Feng, Le Xiao, Gang Wang

**Affiliations:** 1 National Clinical Research Center for Mental Disorders & Beijing Key Laboratory of Mental Disorders, Beijing Anding Hospital, Capital Medical University, No. 5 Ankang Lane, Deshengmenwai Avenue, Xicheng District, Beijing, 100088 China; 2 Mood Disorders Center, Beijing Anding Hospital, Capital Medical University, Beijing, China; 3 Department of Psychiatry, Capital Medical University, Beijing, China; 4 Center of Depression, Beijing Institute for Brain Disorders, Beijing, China

**Keywords:** Scopolamine, Major depressive disorder, Efficacy, Randomized controlled trial

## Abstract

**Background:**

Major depressive disorder (MDD) is a prevalent and disabling disorder that can lead to heavy individual, familial, and societal burdens. Although pharmaceutical interventions still play an essential role in therapeutic measures, limitations, including effects that are delayed for weeks, are noteworthy. Antidepressants with rapid efficacy and acceptable tolerance have been investigated for many years; rapid antidepressant effects and promising clinical applications have been obtained with intravenous and oral scopolamine. This study aims to evaluate the efficacy of repeated intramuscular scopolamine as an add-on treatment to escitalopram.

**Methods:**

This is a single-center, saline-controlled, double-blind, three-armed, randomized trial. Sixty-six participants diagnosed with MDD will be recruited at Beijing Anding Hospital and randomly assigned to one of three groups: a high-dose intramuscular scopolamine augmentation group; a low-dose intramuscular scopolamine augmentation group; and a placebo control group. Our primary endpoint is improvement in the 17-Item Hamilton Rating Scale for Depression (HRSD17) score from the baseline (at least a 20% reduction). Prespecified secondary endpoints include response rates and remission rates as well as changes in the total or subscale scores between the baseline and week 4.

**Discussion:**

This study will provide the first insight regarding the rapid antidepressant efficacy and tolerability of an intramuscular scopolamine add-on to the usual treatment in Chinese MDD patients. The first discussion concerns whether augmentation can accelerate early antidepressant efficacy. A pilot study of intramuscular scopolamine is performed.

The limitations of this study include its small sample size and it being a single-center study, suggesting the need for further confirmation with trials enrolling larger populations.

**Ethics and dissemination:**

The study protocol and all related materials have been approved by the Institutional Ethics Committee of the Beijing Anding Hospital (No. 2016–106, Beijing, China). The findings will be disseminated through peer-reviewed journals and at national and international conferences.

**Trial registration:**

ClinicalTrials.gov, NCT03131050. Registered on 18 April 2017.

**Electronic supplementary material:**

The online version of this article (10.1186/s13063-018-3132-3) contains supplementary material, which is available to authorized users.

## Background

Major depressive disorder (MDD) is a prevalent and disabling condition with a high frequency of non-recovery and recurrence, leading to substantial mortality and morbidity [1–3]. According to the new World Health Organization (WHO) report, the proportion of the global population with depression is estimated to be 4.4% in 2015. In China, > 54 million people (4.2% of the population) suffer from depression [4]. Although antidepressants are the main treatment for MDD, biweekly doses are the minimum requirement for full treatment to be efficacious. Despite the availability of a wide range of antidepressant drugs, clinical studies have indicated that approximately one-third of patients with MDD fail to respond to first-line antidepressant treatment, even if the drugs are used at adequate dosages and durations. Thus, there is an urgent need to develop novel and improved drugs to treat depression [5].

Clinical studies have identified two different classes of drugs with rapid antidepressant action in patients with depression: ketamine, a non-competitive glutamate N-methyl-D-aspartate (NMDA) receptor antagonist [6–8]; and scopolamine, a non-selective acetylcholine muscarinic receptor antagonist [9–12]. Scopolamine has been shown to produce rapid and robust antidepressant effects on current MDD and bipolar disorder patients [9, 13].

In 1991, Gillin discussed the effect of intramuscular injections of scopolamine on depression. In that study, depressive symptoms appeared to improve, but the effect was not significant [13]. In 2006, Furey and Drevets conducted a series of randomized, controlled, double-blind, and crossover clinical trials to determine the antidepressant effects of scopolamine. The results showed that both depressed and bipolar patients demonstrated a rapid and consistent reduction in their Montgomery-Asberg Depression Rating Scale (MADRS) scores by three days after the first infusion. At the end of the trial, nearly two-thirds of the patients improved markedly, in whom half of the symptoms disappeared completely, and the improvement effects lasted for two weeks after the last infusion [9, 11, 12, 14]. In addition to intramuscular and intravenous injections, the antidepressant effect of oral scopolamine has also been discussed; the researchers found that scopolamine and citalopram had an acquired combined effective rate in patients with depression and that citalopram elicited no significant difference regarding the onset time of the antidepressant effects [14, 15].

Previous studies of intramuscular, intravenous, and oral scopolamine have shown some antidepressant effects that have promising clinical applications, but those studies focused on intravenous scopolamine; this study will use the method of intramuscular injections. Compared with intravenous injections, the use of intramuscular injections for the treatment of patients is more convenient and has better acceptability. Although intramuscular injections of scopolamine were shown to be effective in only one of the previous studies, there were limitations in that study including the small number of cases and gender bias [13]. Therefore, we need to improve the program to further explore the efficacy of intramuscular injections.

Due to the lack of a known effective dose of intramuscular scopolamine in previous studies, we designed a dose-finding study using a scopolamine dose range that was previously associated with cognitive effects but without toxic effects such as delirium. In reference to the static drops in scopolamine antidepressant effective doses and to the recommended dose range of scopolamine injection specification, we chose a high-dose group (0.6 mg/day) and a low-dose group (0.3 mg/day). To ensure that the drug clearance was complete before the next injection, we designed the trial protocol to include morning and afternoon intramuscular injections each time; the treatment interval was 6 h.

Most of the trials were crossover studies and only used a single intravenous injection, limiting the generalizability of the findings. Previous studies have shown a rapid antidepressant effect of scopolamine; therefore, we added antidepressant treatment to see if scopolamine could accelerate the onset of antidepressant drugs. Our team’s research showed that single-dose intravenous ketamine augmentation by escitalopram was safe and effective in the treatment of severe MDD, showing the promise of speeding up early oral antidepressant efficacy [16]. To date, the effects of add-on scopolamine on currently available antidepressants have not been examined and it remains unknown whether concurrent initiation or oral antidepressant treatment with intramuscular scopolamine may increase antidepressant efficacy. Taking into account that the antidepressant effect of scopolamine is fast but not sustained, the focus of our study is on how to use scopolamine’s antidepressant effect to help patients with depression symptoms improve in the early stage. Therefore, we aimed to determine the antidepressant effects and safety of low-dose or high-dose single intramuscular scopolamine infusions, which were combined with escitalopram initiation in MDD.

## Objectives

This study is designed to verify whether the augmentation of intramuscular scopolamine may accelerate the efficacy of antidepressant treatment.

## Methods

### Study design

The trial is a single-center, double-blind, three-arm randomized trial with four-week follow-ups at Beijing Anding Hospital, which is affiliated with Capital Medical University. The protocol is reported as following the SPIRIT checklist (see Additional file 1). The actual study start date was 15 March 2017 and the completion date is 31 August 2018.

### Participants

All patients diagnosed at the outpatient clinics of Beijing Anding Hospital between 1 February 2017 and 31 August 2018, will be identified as potential participants and be invited to participate in this study.

#### Inclusion criteria

1. Aged 18–45 years;
2. diagnosis of MDD according to the Diagnostic and Statistical Manual of Mental Disorders, Fourth Edition (DSM-IV) criteria;
3. a total score of the 17-Item Hamilton Rating Scale for Depression (HRSD17) ≥ 20;
4. duration of the index episode is ≥ 4 weeks.

#### Exclusion criteria

1. Current enrollment in, or discontinuation within the last 30 days from, a clinical trial involving off-label use of an investigational drug;
2. other disorders in DSM Axis I;
3. organic mental diseases, including mental retardation;
4. history of any clinically significant disease, including cardiovascular, hepatic, renal, respiratory, hematologic, endocrinologic, or neurologic disease or clinically significant laboratory abnormalities that are not stabilized or are anticipated to require treatment during the study;
5. exposure to different formulations and generic agents of investigational drugs during the three months before screening;
6. a positive pregnancy test or breast feeding;
7. women of childbearing potential without appropriate birth control measures;
8. antipsychotics or mood stabilizers within the five days before screening;
9. allergy to or lack of response to escitalopram;
10. electroconvulsive therapy (ECT) or modified ECT within the three months before screening;
11. hypersensitivity to anticholinergic agents;
12. smoking;
13. significant risk of suicide, as evidenced by scoring 3 or 4 for HRSD17 Item 3, and risk of self-harm behaviors as ascertained by the investigator after inquiry.

### Interventions

Individuals are equally and randomly allocated to three treatment regimens groups that are different only for the first three days: (1) the high-dose group receives 0.3 mg of intramuscular scopolamine twice daily; (2) the low-dose group receives 0.3 mg of scopolamine in the morning and saline in the afternoon; and (3) the saline-controlled group is injected with intramuscular saline twice daily. After the first three days of add-on injections, the participants receive monotherapy with a fixed dose of oral escitalopram for the remaining 25 days. Injections are administered at 09:00 and 15:00 by the research nurse.

All the individuals stay in the emergency wards during the first three days and receive clinical visits on the 4th, 7th, 14th, and 28th day from the first injection.

**Fig. 1:**
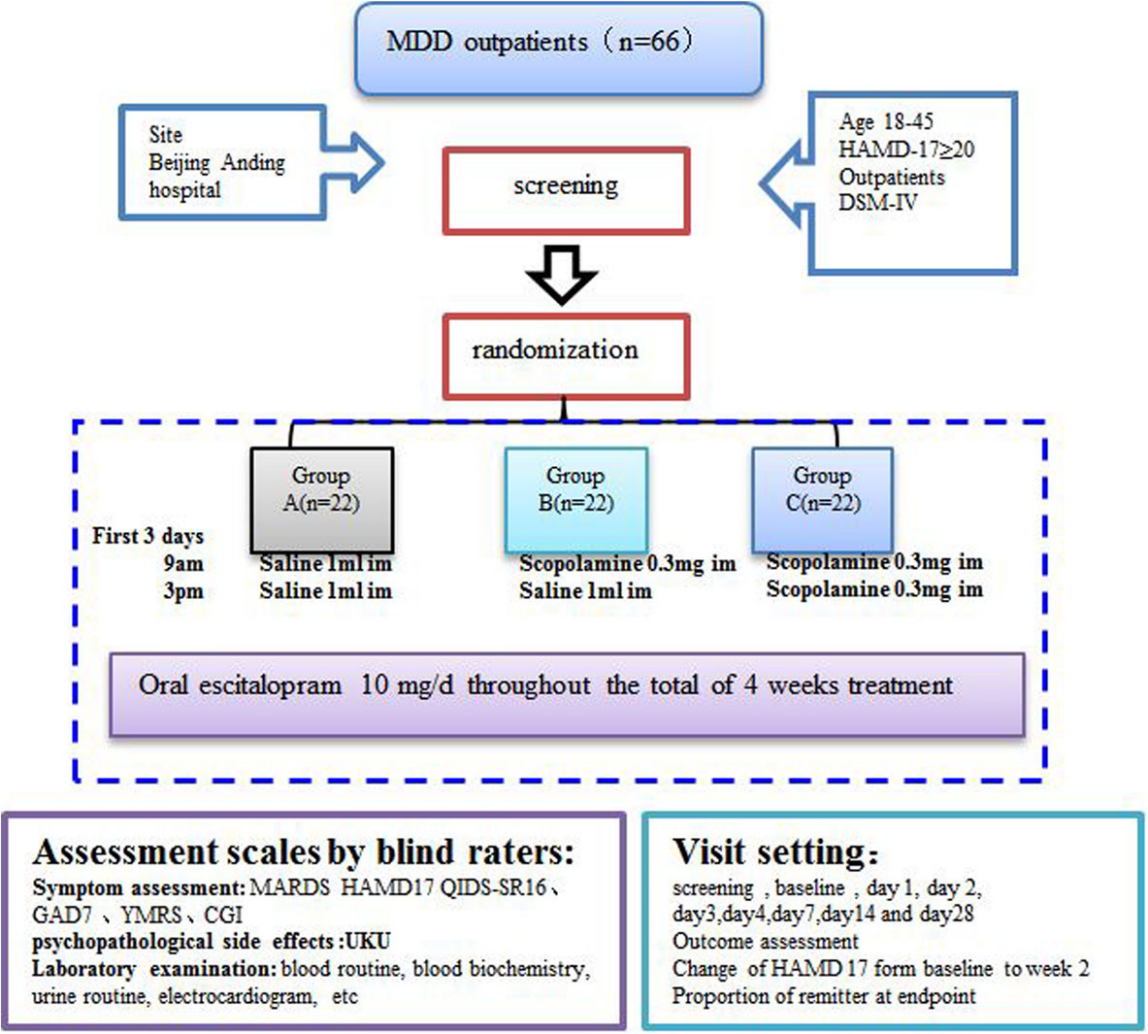
Study *flow chart*

See “Fig. 1 Study flow chart” for a detailed overview of the research procedure.

#### Drug/therapy combination

Drug or therapy combinations are permitted in cases of preset conditions as follows: (1) if participants suffer from insomnia, sleep aides (zolpidem [≤ 10 mg daily], zopiclone [≤ 7.5 daily], or zaleplon [≤ 10 mg daily]) are permitted for up to seven days total throughout the study and in any case of ineffectiveness, a switch can only occur between those on the list; and (2) administration of lorazepam is permitted for short-term management of treatment for adverse events (AEs), such as anxiety and agitation, up to a maximum of 3 mg/day. Benzodiazepines must not be administered within the 8 h before scheduled efficacy and safety assessments.

### Outcomes

The primary outcome for this study is improvement of the HRSD17 score from the baseline to 28 days (at least a 20% reduction). Secondary outcomes are response rate (a 50% decrease in HRSD17 score from baseline), remission rate (HRSD17 score ≤ 7), changes in HRSD17, MADRS, Quick Inventory of Depressive Symptomatology Self Report 16-Item (QIDS-SR16), Generalized Anxiety Disorder 7-Item (GAD-7), Young Mania Rating Scale (YMRS), and Clinical Global Impression (CGI) Scale scores throughout the trial.

The Chinese version of the HRSD17 has satisfactory psychometric properties in terms of validity and reliability. The internal reliability and the validity are good (Cronbach alpha = 0.714; *r* = − 0.487) [17]. The scale is widely used to measure the efficacy of antidepressant medication in clinical trials, as HRSD17 is more sensitive to change on retesting [18, 19]. The MADRS is an interviewer-rated instrument, comprising 10 items, each measured on a 6-point scale (scores range from 0–60 with higher scores depicting greater symptom severity). It has been found to be a robust and psychometrically sound measure of depressive symptomatology [20]. CGI was used to assess change in symptoms from baseline to endpoint. QIDS-SR16, a measure of depressive symptom severity derived from the Inventory of Depressive Symptomatology, has highly acceptable psychometric properties (Cronbach alpha = 0.70; *r* = 0.61) which supports the usefulness of this brief rating of depressive symptom severity in both clinical and research settings [21]. There are 16 items in the adult versions of the QIDS-SR16 measuring the nine criterion symptom domains (sleep, sad mood, appetite/weight, concentration/decision making, self-view, thoughts of death or suicide, general interest, energy level, and restlessness/agitation) that define a major depressive episode according to the DSM-IV. GAD-7 is a self-reported questionnaire for screening and for measuring the severity of generalized anxiety disorder. Cronbach’s α coefficient and an area under the receiver operating curve for the GAD-7 are 0.888 and 0.974, respectively [22]. YMRS is an 11-item multiple choice diagnostic questionnaire used to evaluate manic symptoms. The CGI was developed for use in clinical trials to provide a brief, stand-alone assessment of the clinician’s view of the patient’s global functioning before and after initiating a study medication. The CGI comprises two companion one-item measures evaluating the following: (1) severity of psychopathology on a scale of 1 to 7; and (2) change from the initiation of treatment on a similar 7-point scale.

### Sample size

According to the published research and clinical expectations, we estimated the median time from the baseline to early improvement (at least a 20% reduction of HRSD17 score) of scopolamine plus escitalopram is 6.4 days and the four-week remission rate is 92.3%. The median time from the baseline to early improvement of placebo plus escitalopram treatments is 26.5 days and the four-week remission rate is 57.1%. Considering the time to early improvement, which is fitted to an exponential distribution, and assuming a power of 80% with a two-sided significance of 0.01, a minimal sample size of 54 was calculated using survival (time to event) modules. A common method for testing the proportional hazards assumption is to include a time interaction term to determine if the hazard ratio changes over time, since time is often the culprit behind non-proportionality of the hazards. Evidence that the group*time interaction term is not zero is evidence against proportional hazards. Assuming an attrition of 10%, at least 66 participants are planned.

### Recruitment

Patients will be mainly recruited via referral from psychiatrists in the outpatient department. At the screening visit, the trained research investigators and blind raters collect and make judgments regarding eligibility. The Chinese version of the MINI is used to confirm that the DSM-IV criteria for MDD are met and to assess the exclusion criteria. Vital signs, weight, height, physical examination, ECGs, and clinical safety laboratory tests are taken sequentially.

### Randomization, allocation concealment, and blinding

Eligible individuals will be randomized in a 1:1:1 ratio to one of the treatment regimens and a blocked randomization protocol will be designed to allocate an equal number of individuals to each group. The randomization code will be created using a blocked randomization procedure in SAS 9.4 (SAS Institute Inc., Cary, NC, USA) by a statistician who is not involved in the study. The block size is nine. All parameters will be maintained by statistician. According to the protocol, the research assistants who do not participate in the storage or allocation of the investigational drug will pack and label the investigational drug. Each package contains all the dosages of the investigational drug needed for the whole study. The eligible patients will receive a subject ID in order and be assigned drug package. The participants and raters will be blind to treatment allocation. If serious AEs, overdose, or severe drug interactions occur, blindness will be broken by PI with an emergency envelope for the purpose of guiding follow-up treatment to the allocation.

### Data collection and management

**Fig. 2:**
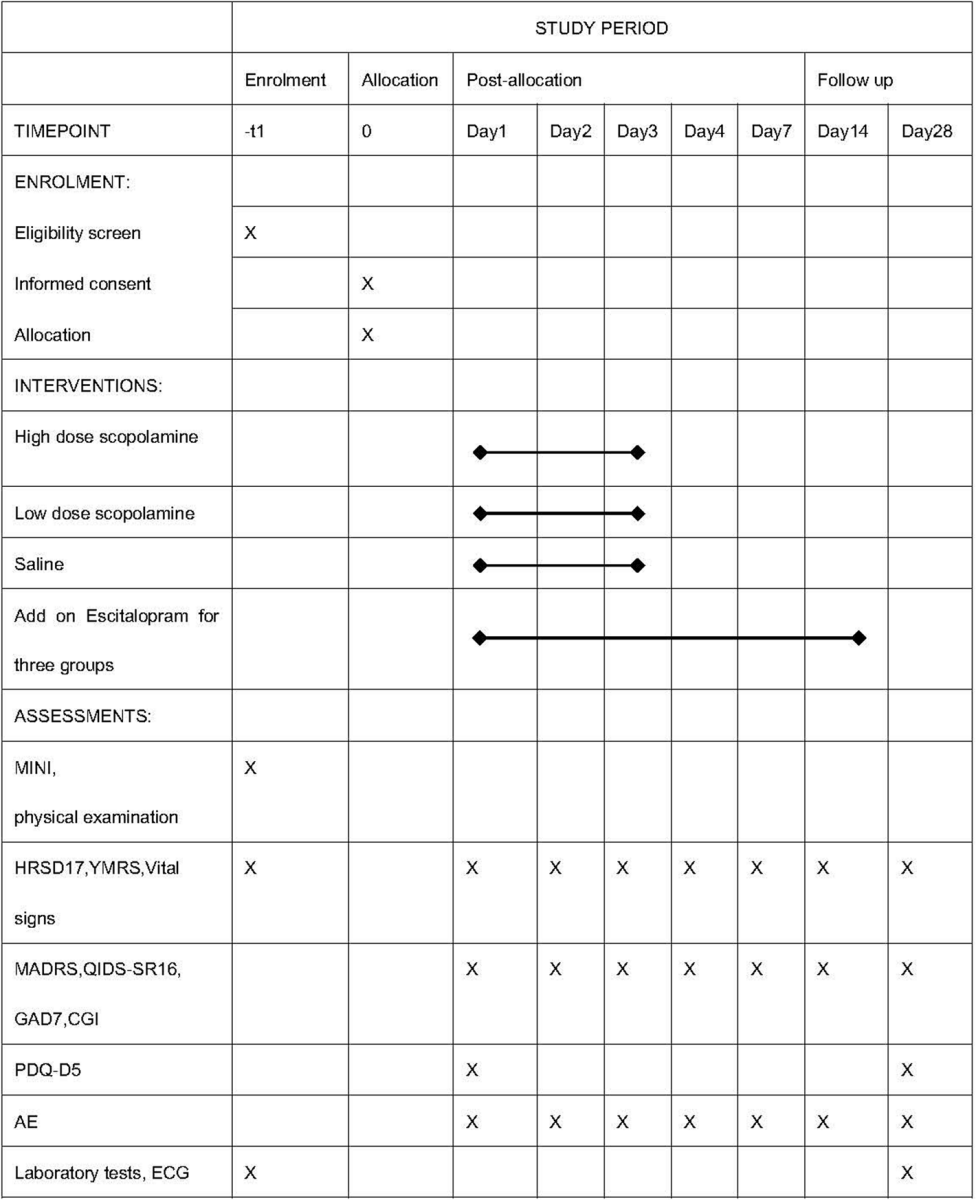
Schedule of enrolment, intervention, and assessments (Standard Protocol Items: Recommendations for Interventional Trials (SPIRIT) diagram)

After enrollment, medical histories are collected via the case report form (CRF). Depressive symptoms, anxiety symptoms, and cognitive and functional impairments are assessed via the standardized Chinese version of the HRSD17, MARDS, QIDS-SR16, GAD7, CGI, SDS, and Perceived Deficits Questionnaire 5-Item (PDQ-D5) scales, respectively. The measures, participants, methods, assessment times, and administrators of the data collection are listed in Fig. 2. The data collected from medical records, dedicated scale manuals, well-designed questionnaires, laboratory/ECG test reports, and procedure records are defined as source data, and the data are structured and transcribed into CRF. After being reviewed by a quality control group and the PI, CRFs will be transferred to the data manager. Basic formats and logic checks will be performed by the data manager. Questionable data will be marked and the investigator will be informed to answer and modify the data. The database will be locked and stored after all questionable data is modified. Backup files for the database will be required to prevent defects. All transmissions of files will be recorded with signatures and be preserved following the good clinical practice (GCP) guidelines.

The research team includes qualified research staff authorized by the principle investigator ahead of any activity, investigators, independent raters, research nurses, and clinical research associate. The raters are continuously trained and tested by two senior raters and the inter-reliability of the primary outcome measure (HRSD17) is audited for each rater.

### Statistical analysis

All collected data will be analyzed using the SAS Statistical Package (version 9.4; (SAS Institute Inc., Cary, NC, USA). The sample characteristics will be compared using chi-square tests for categorical variables, with Fisher’s Z transformation when needed, and t-tests for normal quantitative variables or the Mann–Whitney U test for non-normal quantitative variables.

Efficacy analyses will be based on an intent-to-treat approach, corresponding to the full-analysis set (FAS), comprising all patients assigned to any treatment group and who have a valid baseline assessment of the primary efficacy outcome variables (HRSD17 total scores). Kaplan–Meier survival analyses will be used to calculate the estimated time from the baseline to early improvement. The Cox proportional hazards regression model will be applied to compare the estimated time to early improvement between the three groups, while controlling for covariates such as age, sex, and duration of illness. Secondary analyses will be performed to assess changes from baseline to each visit in terms of HRSD17, YMRS, CGI, and SDS total scores, using a mixed model of repeated measures (FAS, Mixed-Effect Model Repeated Measure model), with treatment as the fixed factor and baseline scores and age as covariates. A *P* value < 0.05 is considered as statistically significant. The statistical analysis protocol is validated before the interim analysis.

### Monitoring

Standardized terminology will be used to categorize AEs. The number and proportion of each AE will be summarized according to the system organ class and the preferred term. The PDQ-D5 is administered to assess perceived cognitive deficits from the patient’s perspective.

Interim analysis will be conducted when the study sample reaches 40% of the entire sample to re-evaluate sample size, in reference to the therapeutic effect. If the treatment effect is smaller than expected, we may need to increase the sample size. Blindness will be maintained during the interim analysis. An independent statistician, who is separate from the research team, will perform the interim analysis. The O’Brien-Fleming method [23] will be used to control for type Ι errors. Safety analyses will be used to evaluate hazards with the intention of discontinuing the trial to protect the patients. Type, frequency, and severity of AEs are used to assess safety.

## Discussion

With the high morbidity and high risk of depression, we urgently need to explore antidepressant drugs that can be effective in the short term. Although several previous randomized, controlled studies have examined single scopolamine infusion versus placebo for treatment of unipolar or bipolar depression, all but one showed intramuscular injections of scopolamine to be effective. This is the first randomized, double-blind, controlled trial to systematically investigate the effect of a single intramuscular injection of scopolamine as an adjunct to newly initiated treatment in patients with MDD, with the aim of increasing antidepressant efficacy. This research will open up new areas for the development of antidepressants.

### Trial status

Protocol version 1 (1 February 2017); recruitment start date 15 March 2017; initial submission date 4 September 2017; projected recruitment completion data 31 August 2018.

## Additional file

Additional file 1: SPIRIT 2013 Checklist: Recommended items to address in a clinical trial protocol and related documents*. (DOC 116 kb)

## Abbreviations

CGI: Clinical Global Impression
CRF: Case report form
DSM-IV: Diagnostic and Statistical Manual of Mental Disorders, Fourth Edition
ECT: Electroconvulsive therapy
FAS: Full-analysis set
GAD-7: Generalized Anxiety Disorder 7-Item
GCP: Good clinical practice
HRSD17: Hamilton Rating Scale for Depression 17-Item
MADRS: Montgomery-Asberg Depression Rating Scale
MDD: Major depressive disorder
NMDA: N-methyl-D-aspartate
PDQ-D5: Perceived Deficits Questionnaire 5-Item
QIDS-SR16: Quick Inventory of Depressive Symptomatology Self Report 16-Item
YMRS: Young Mania Rating Scale
